# SORBS2: A Molecular Nexus in Multisystem Diseases Through Scaffold‐Mediated Regulation

**DOI:** 10.1111/jcmm.71160

**Published:** 2026-04-27

**Authors:** Qiwei Jia, Yong Zhang

**Affiliations:** ^1^ Department of Hepatobiliary Surgery, Union Hospital Tongji Medical College, Huazhong University of Science and Technology Wuhan China

**Keywords:** cardiovascular diseases, metabolic diseases, neoplasms, nervous system disease, scaffold protein, SORBS2

## Abstract

Sorbin and SH3 Domain Containing 2 (SORBS2), a multifunctional scaffold protein harbouring Sorbin homology (SoHo) and Src homology 3 (SH3) domains, serves as a molecular hub in human diseases by integrating cytoskeletal remodelling, signal transduction and RNA metabolic regulation. This study systematically analyses SORBS2's molecular features, expression regulatory mechanism and disease associations. In oncology, it suppresses metastasis via enhancing the stability of certain mRNAs and immunomodulation yet exhibits oncogenic properties in triple‐negative breast cancer. Cardiovascular manifestations demonstrate dose‐sensitive pathology: deficiency causes arrhythmogenic cytoskeletal disorganization, while overexpression induces β‐tubulin hyper polymerization and ventricular maldevelopment. Epigenetic silencing by miR‐484 exacerbates metabolic liver disease, whereas defective interaction with the large‐conductance Ca^2+^‐activated K^+^ (BK) channel drives diabetic vasculopathy. Neurologically, it modulates synaptic remodelling and neuroinflammatory pathways. Functioning as a signalling nexus, SORBS2 interconnects chronic inflammation, oxidative stress and metabolic dysfunction, supporting ‘one target for multiple diseases’ strategy. Future research requires integration of single‐cell omics, Artificial intelligence (AI)‐based drug design and epigenetic editing for clinical translation.

## 1 Introduction

**FIGURE 1:**
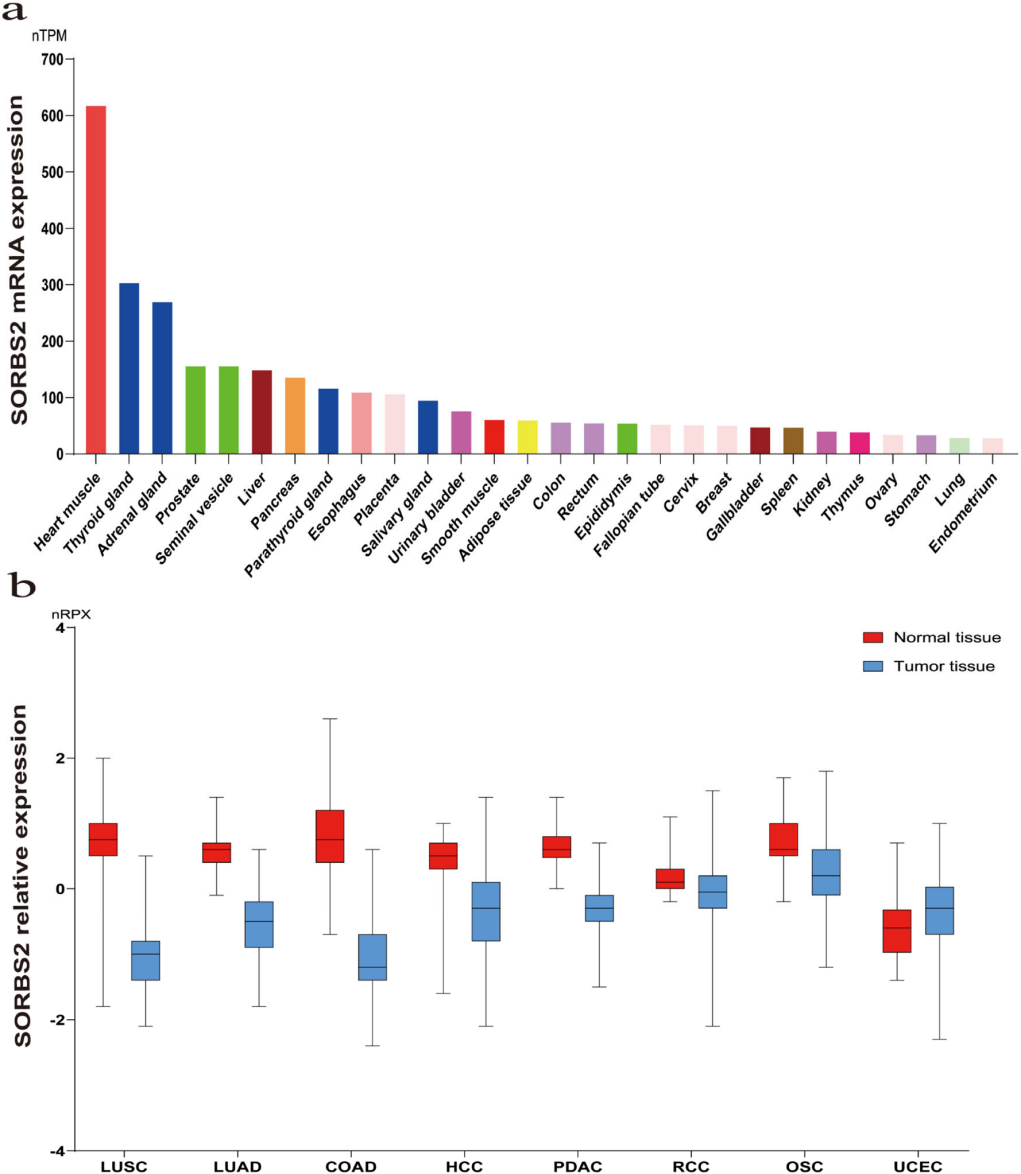
The relative expression levels of SORBS2 mRNA in normal tissues and organs and the alterations in its relative protein expression levels in disease states. (a) The relative expression levels of SORBS2 mRNA in normal tissues and organs, arranged from highest to lowest. (b) A comparison of the relative protein expression levels of SORBS2 in common malignant tumours versus normal tissues, where SORBS2 expression is decreased in the majority of tumours, while exhibiting a modest increase in a minority of tumours. COAD, Colon adenocarcinoma; HCC, Hepatocellular carcinoma; LUAD, Lung adenocarcinoma; LUSC, Lung squamous cell carcinoma; nRPX is calculated as log_2_(intensity), derived from glodance measurements at the protein level; nRPX, normalized Protein eXpression; nTPM, normalized protein‐coding transcripts per million; OSC, Ovarian serous carcinoma; PDAC, Pancreatic ductal adenocarcinoma; RCC, Renal cell carcinoma; UCEC, Uterine corpus endometrial carcinoma.

Scaffold proteins are a class of proteins that play critical roles in cellular signal transduction and other biological processes. Their primary function is to organize specific signalling complexes by physically binding multiple signalling molecules (e.g., kinases, phosphatases and receptors) thereby regulating the efficiency and specificity of intracellular signal transmission [1]. SORBS2, a scaffold protein encoded by the *SORBS2* gene (also known as *ARGBP2*, for Arg kinase‐binding protein 2, located on human chromosome 4q35.1), belongs to the SORBS protein family. This family includes SORBS1 (CAP/Ponsin) and SORBS3 (Vinexin), all characterized by possessing one N‐terminal SoHo domain and three C‐terminal SH3 domains [2]. SORBS2 is widely distributed in muscular tissues (e.g., skeletal muscle, cardiac muscle), brain tissue, liver and vascular endothelial cells, where it exhibits high expression levels (Figure 1a). Its subcellular localization is highly dynamic, predominantly enriched at focal adhesions, within the cytoplasm and at the plasma membrane, where it exerts essential biological functions [3, 4, 5].

First identified in 1997 as a scaffold protein associated with Abl/Arg non‐receptor tyrosine kinases, nearly three decades of research have established that aberrant expression or dysfunction of SORBS2 is implicated in diverse pathological processes [4]. In cardiovascular diseases, SORBS2 deficiency contributes to structural heart disease, arrhythmias and heart failure [6]. In malignancies, SORBS2 exhibits dual roles, primarily suppressing tumour progression while paradoxically promoting malignant phenotypes under specific conditions [7, 8]. In metabolic disorders, SORBS2 participates in the pathogenesis of diabetes, obesity, and non‐alcoholic fatty liver disease (NAFLD) [9, 10, 11]. These findings underscore SORBS2's pivotal role as a molecular hub in disease networks. This review will systematically elucidate the pathological roles of SORBS2 in cancer, cardiovascular diseases, metabolic disorders and neuropsychiatric conditions, beginning with its molecular characteristics and regulatory mechanisms. Furthermore, it will analyse the common mechanisms by which SORBS2 connects multisystem diseases as a ‘molecular hub’ and explore its clinical translational potential.

## 2 The Functions and Regulatory Mechanisms of SORBS2

### 2.1 Structural Basis and Molecular Diversity

SORBS2 is a multifunctional scaffold protein whose structural features and splice variant diversity underpin its broad biological roles in cells. The N‐terminus of SORBS2 contains a SoHo domain, which targets lipid rafts—a feature critical for intracellular communication—while the C‐terminus harbours three SH3 domains that recognize proline‐rich sequences [12, 13]. Additionally, the SORBS2 protein sequence includes post‐translational modification sites, such as phosphorylation and ubiquitination sites, regulated by kinases or phosphatases to modulate protein localization or activity [13]. SORBS2 has at least five splice variants, including SORBS2α, SORBS2β, SORBS2γ, SORBS2δ and the neuronal splice variant nSORBS2 [4, 6, 14, 15]. Among these, the α and γ variants are larger isoforms retaining both the SoHo domain and three SH3 domains, whereas the β and δ variants are shorter forms lacking the SoHo domain. nSORBS2, a neuron‐specific protein localized to cell bodies, neurites and synapses, contains the SoHo domain, a zinc finger motif and three SH3 domains. It contributes to postsynaptic density formation and cytoskeletal‐synaptic component linkage by interacting with synaptic proteins such as synapse‐associated protein 90/postsynaptic density‐95‐associated protein (SAPAP) [14].

### 2.2 Expression Regulation

**FIGURE 2:**
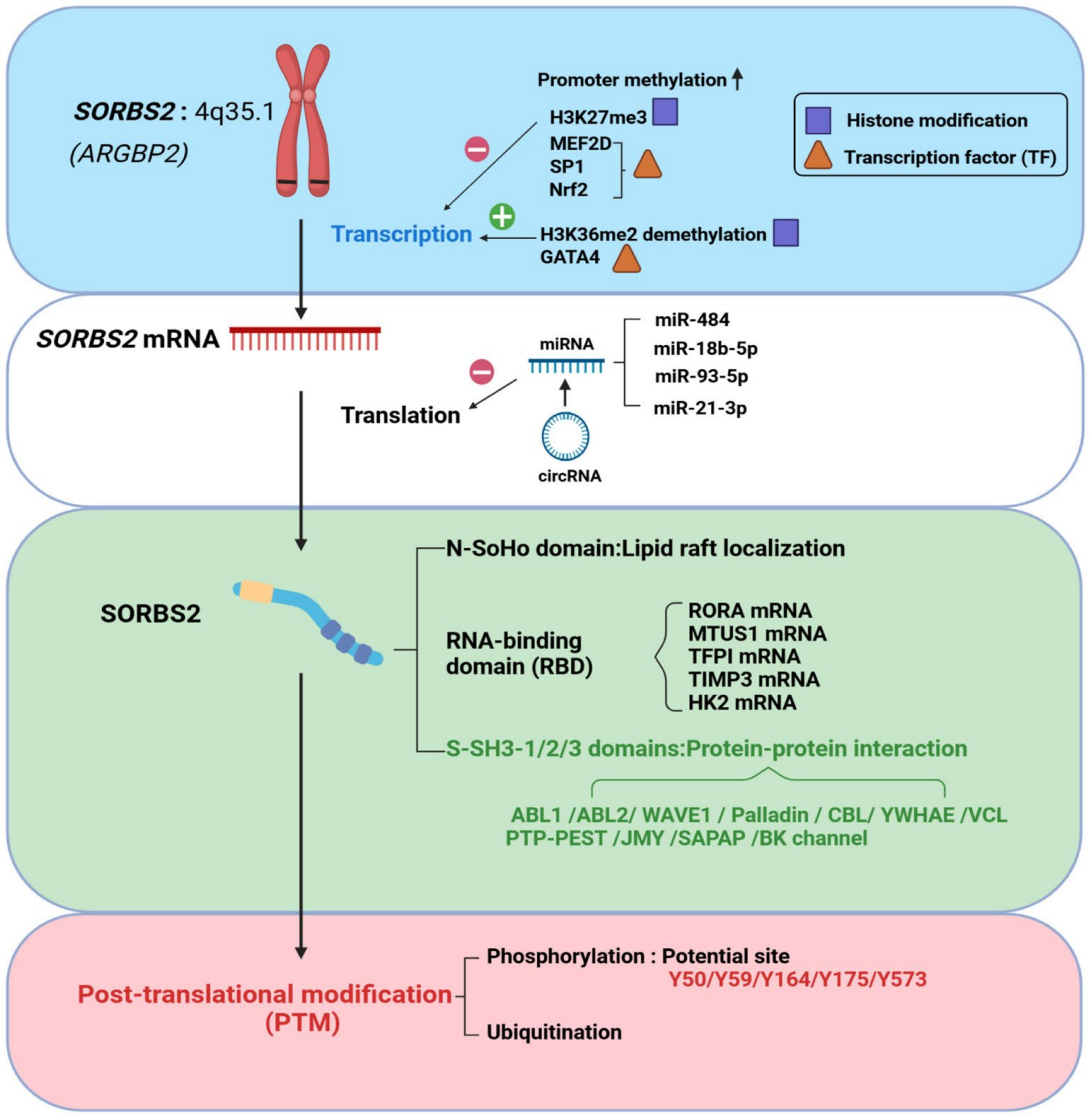
The expression regulation of the SORBS2 gene and the functional roles of the SORBS2 protein. The transcription process of SORBS2 can be regulated by promoter methylation, histone modifications and transcription factors; during its translation process, it is also influenced by miRNAs. SORBS2 can interact with other proteins or mRNAs through its SoHo domain, SH3‐1/2/3, and RNA‐binding domain, and produce biological effects. After translation is completed, SORBS2 is also subject to chemical modifications such as phosphorylation and ubiquitination, which are core to its functional regulation.

The expression and function of SORBS2 are regulated through multi‐layered, precise mechanisms (Figure 2). At the transcriptional level, the methylation status of its promoter region dynamically modulates gene expression. For instance, in adipose tissue, promoter methylation of SORBS2 correlates negatively with its expression [11]. Studies have shown that heat shock transcription factor 1 (HSF1) directly binds MORC family CW‐type zinc finger 2 (MORC2), and together they target the SORBS2 promoter region, recruiting EZH2 (an H3K27me3‐specific methyltransferase) to promote histone H3 lysine 27 trimethylation (H3K27me3), resulting in chromatin compaction and transcriptional repression of SORBS2 [16, 17]. Additionally, non‐coding RNAs (e.g., miR‐484, miR‐18b‐5p and miR‐93‐5p) suppress SORBS2 translation by targeting its mRNA's 3′UTR, significantly downregulating its expression [10, 18, 19]. Post‐translational modifications (PTMs)—phosphorylation and ubiquitination—are central to SORBS2 functional regulation. SORBS2 serves as a substrate for ABL family kinases and contains five tyrosine residues (Y50, Y59, Y164, Y175 and Y573) conforming to the c‐Abl kinase substrate consensus sequence (YXXP), which are potential phosphorylation sites [4]. Furthermore, SORBS2 harbours a serine/threonine‐rich region that may also undergo phosphorylation, though the specific kinases and functional implications require further investigation [4]. Oligomerization of SORBS2 constitutes another regulatory mechanism. Research demonstrates that SORBS2 forms oligomers via SH3‐B domain‐mediated binding to its own P1 proline cluster, with this self‐assembly dynamically modulating its interaction with signalling proteins such as c‐Abl and PTP‐PEST [20]. Collectively, these mechanisms respond to intra‐ and extracellular signals (e.g., mechanical stress, growth factors or DNA damage), finely tuning SORBS2 abundance, localization and functional activity, thereby influencing cell adhesion, migration and disease progression.

### 2.3 Scaffolding Mechanisms of SORBS2

As a multi‐domain scaffold protein, SORBS2 coordinates diverse cellular processes by simultaneously binding multiple interaction partners through its three SH3 domains and N‐terminal SoHo domain. This scaffolding function extends beyond static structural features to dynamically integrate signal transduction, cytoskeletal organization and RNA metabolism.

First, as a signalling scaffold, SORBS2 assembles multiprotein complexes that regulate signal transduction. Its SH3 domains directly bind proline‐rich motifs in various signalling proteins, including the non‐receptor tyrosine kinases c‐Abl and Arg, serving as both an adaptor and substrate to potentially modulate kinase activity and substrate localization [4]. It also interacts with Cbl proteins, thereby participating in ubiquitination‐associated signalling pathways [21]. This scaffolding function is dynamically regulated by phosphorylation: phosphorylated SORBS2 enhances binding to c‐Abl but inhibits interaction with the phosphatase PTP‐PEST [5]. Moreover, phosphorylated SORBS2 facilitates Cbl recruitment, which mediates ubiquitination and proteasomal degradation of both SORBS2 and c‐Abl, representing a key negative feedback mechanism [21].

Second, as a cytoskeletal scaffold, SORBS2 links signalling complexes to the actin cytoskeleton and maintains mechanical integrity. Through its SoHo domain, it binds α‐actinin, and via its first SH3 domain, it interacts with palladin, forming ternary complexes that target cardiac Z‐discs and non‐muscle stress fibre dense bodies [3]. These interactions stabilize the actomyosin network and enhance its crosslinking, thereby suppressing cell migration [22]. SORBS2 further modulates actin dynamics by regulating Abl/Arg kinase‐mediated phosphorylation of cytoskeletal components [3]. In the cardiovascular system, this scaffolding function extends to ion channel regulation: SORBS2 interacts with voltage‐gated Na^+^ channels (Nav1.5), L‐type Ca^2+^ channels (Cav1.2) and large‐conductance Ca^2+^‐activated K^+^ (BK) channels in vascular smooth muscle cells, modulating their expression and function [6, 23]. In the male reproductive system, the JMY‐SORBS2‐α‐actinin1 complex regulates actin crosslinking and anchoring to maintain blood‐testis barrier integrity and dynamic cell junctions essential for spermatogenesis [24].

Third, as an RNA metabolic scaffold, SORBS2 directly binds target mRNAs through its RNA‐binding domain, forming ribonucleoprotein complexes that influence mRNA stability and subsequent gene expression [25, 26, 27]. This function enables SORBS2 to post‐transcriptionally regulate genes involved in tumour suppression (e.g., RORA, MTUS1, TFPI and TIMP3) and metabolism (e.g., HK2), thereby exerting biological effects independent of its protein–protein interaction network [7, 27, 28, 29, 30].

Collectively, these scaffolding mechanisms enable SORBS2 to integrate signal transduction, cytoskeletal dynamics and post‐transcriptional gene regulation, positioning it as a central hub coordinating cellular responses to diverse stimuli.

### 2.4 Molecular Mechanisms and Disease: Pathological Implications of Scaffold Dysfunction

The preceding sections have established that SORBS2 functions as a multi‐dimensional scaffold protein, integrating signal transduction, cytoskeletal dynamics and RNA metabolism through its distinct structural domains and regulatory mechanisms (Table 1). Under physiological conditions, these interconnected functions maintain cellular homeostasis by coordinating cellular responses to mechanical stress, growth factors and other extracellular stimuli. However, under pathological conditions, destabilization of this finely tuned system—triggered by epigenetic silencing (e.g., promoter hypermethylation) or aberrant post‐translational modifications (e.g., dysregulated phosphorylation)—can initiate cascading effects: aberrant microtubule polymerization in cardiomyocytes leads to structural cardiac remodelling; accelerated mRNA degradation within the tumour microenvironment drives invasive metastatic phenotypes; mitochondrial dysfunction in metabolic organs forms a vicious cycle with insulin resistance. The following sections will delineate the specific roles and mechanisms of SORBS2 in malignancies, cardiovascular diseases, metabolic disorders and neuropsychiatric conditions.

**TABLE 1:** Summary of SORBS2 molecular interactions and functional consequences.

Interacting molecule	Binding domain	Functional outcome	Associated disease(s)	References
c‐Abl	SH3	Regulates kinase activity & substrate localization; enhanced binding upon phosphorylation	Cancer metastasis, leukaemia	[4, 16, 31]
PTP‐PEST	SH3	Dephosphorylates SORBS2; binding inhibited by SORBS2 phosphorylation	Pancreatic cancer cell migration	[5, 30]
Cbl	SH3	Mediates ubiquitination and degradation of SORBS2 and c‐Abl	Signal transduction regulation	[16]
Shank3/CYFIP2	SH3	SH3 interacts with synaptic scaffolding proteins	Autism, Fragile X syndrome	[32, 33, 34]
Palladin	SH3	Targets dense bodies of stress fibres	Cytoskeletal organization	[3]
WAVE1	SH3	Regulates Actin polymerization; loss leads to WAVE1 hyperactivation	Tumour metastasis	[30]
CIP4	SH3	Disrupts SORBS2‐c‐Abl interaction	Tumour metastasis	[30]
BK channel (α/β1)	SH3/SoHo	Maintains channel Ca^2+^ sensitivity and vasodilatory function	Diabetic vasculopathy	[18]
α‐Actinin	SoHo	Maintains structural stability of Z‐discs and stress fibres	Cardiomyopathy, suppression of cell migration	[3, 17, 35]
YWHAQ	—	Suppresses cardiomyocyte cell cycle progression	Left ventricular non‐compaction	[36]
RORA mRNA	RNA‐binding domain	Stabilizes mRNA, inhibiting EMT	Hepatocellular carcinoma (HCC)	[7]
MTUS1 mRNA	RNA‐binding domain	Stabilizes mRNA, inhibiting microtubule depolymerization	Clear cell renal cell carcinoma	[22]
TFPI mRNA	RNA‐binding domain	Stabilizes mRNA, reversing EMT and suppressing metastasis	Bladder cancer	[28]
TIMP3 mRNA	RNA‐binding domain	Inhibits MMP‐mediated ECM degradation	Oesophageal squamous cell carcinoma	[29]
HK2 mRNA	RNA‐binding domain	Stabilizes mRNA, enhancing glycolysis and promoting migration	Trophoblast disorders	[37]

## 3 The Role of SORBS2 in Human Diseases

### 3.1 Malignancies

**FIGURE 3:**
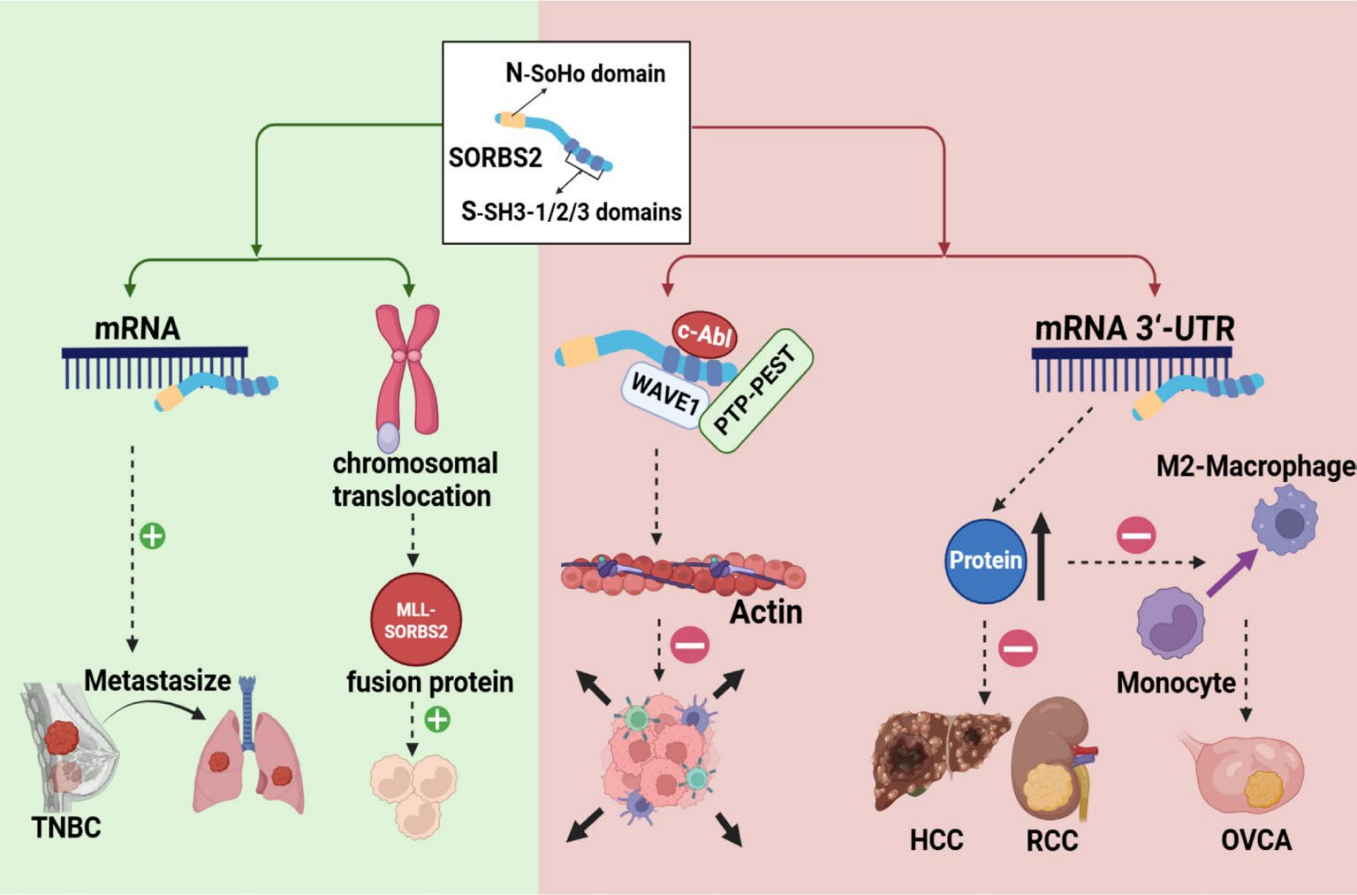
The function of SORBS2 in malignant tumours. SORBS2 primarily exerts tumour‐suppressive effects in malignant tumours, but exhibits tumour‐promoting properties in certain cancers. Its principal mechanisms include: SORBS2 binds to target mRNAs via its RNA‐binding module, enhancing their stability to regulate gene expression, thereby mediating either tumour suppression or promotion; SORBS2 interacts via its SH3 domain with proteins such as c‐Abl, WAVE1 and PTP‐PEST, consequently inhibiting tumour metastasis.

Recent studies have demonstrated that SORBS2 exhibits complex biological functions in various malignancies, acting both as a tumour suppressor and, under specific conditions, a promoter of malignant phenotypes (Figure 3). This functional duality likely arises from its tissue‐specific expression, splice isoform diversity and dynamic regulation of molecular interaction networks. The following sections will systematically elaborate on the dual roles of SORBS2 in malignancies and their underlying molecular mechanisms.

#### 3.1.1 Suppression of Tumour Initiation and Progression

##### 3.1.1.1 mRNA Stability Regulation

SORBS2 directly targets the 3′UTR of tumour suppressor gene mRNAs via its RNA‐binding domain (e.g., C2H2 zinc finger domain), enhancing their stability and suppressing pro‐oncogenic signalling pathways. In hepatocellular carcinoma (HCC), SORBS2 binds to the 3′UTR of RORA mRNA, reducing its degradation and thereby inhibiting the epithelial‐mesenchymal transition (EMT) process, which diminishes tumour cell migration and invasive capacity [7]. A similar mechanism is observed in clear cell renal cell carcinoma, where SORBS2 binds MTUS1 mRNA, stabilizing it to inhibit phosphorylation of the microtubule‐depolymerising protein KIF2C, thereby blocking microtubule dynamics‐driven cancer cell metastasis [27]. In bladder cancer, SORBS2 elevates tissue factor pathway inhibitor (TFPI) protein levels by binding the 3′UTR of TFPI mRNA, reversing EMT and suppressing lung metastasis. Concurrently, the transcription factor SP1 negatively regulates SORBS2 expression by binding its promoter, forming an SP1‐TFPI regulatory axis [28]. Furthermore, in oesophageal squamous cell carcinoma, SORBS2 stabilizes the metalloproteinase inhibitor TIMP3 mRNA, inhibiting matrix metalloproteinase (MMP)‐mediated extracellular matrix degradation and thus impeding tumour invasion [29]. These findings indicate that SORBS2's RNA‐binding functionality constitutes a core mechanism underlying its tumour‐suppressive effects across cancer types.

##### 3.1.1.2 Cytoskeletal and Adhesion Regulation

Under physiological conditions, the SH3 domain of SORBS2 forms a ‘phosphorylation‐dephosphorylation’ regulatory module by simultaneously binding WAVE1, PTP‐PEST and c‐Abl, thereby controlling cytoskeletal dynamics. However, in pancreatic cancer cells, loss of SORBS2 leads to WAVE1 hyperactivation and PTP‐PEST dysfunction, promoting invasive migration; restoring SORBS2 expression suppresses tumour metastatic potential by inhibiting this pathway [5, 38]. Additionally, overexpression of Cdc42‐interacting protein 4 (CIP4) competitively binds the SH3 domain of SORBS2, disrupting its interaction with c‐Abl and attenuating SORBS2's anti‐migratory function. This highlights the critical role of the SORBS2‐CIP4 dynamic equilibrium in regulating tumour metastasis [38]. In Burkitt lymphoma, homozygous deletion of the chromosomal 4q35.1 region causes C‐terminal truncation of SORBS2, abolishing SH3 domain‐mediated c‐Abl binding and disrupting cytoskeletal dynamics, thereby facilitating lymphoma cell dissemination [39]. Furthermore, in breast cancer, SORBS2 overexpression inhibits proliferation by inducing G1‐phase cell cycle arrest (downregulating Cyclin D1 and cyclin‐dependent kinase 4 [CDK4]) and reduces cell migration and invasion by suppressing RhoA/ROCK pathway‐mediated phosphorylation of myosin light chain (MLC) [40].

In summary, in various epithelial‐derived malignancies, SORBS2 functions as a tumour‐suppressive barrier by stabilizing tumour suppressor gene transcripts through its RNA‐binding domain and maintaining epithelial cell adhesion and polarity via its SH3 domain‐mediated interaction with cytoskeletal proteins such as α‐actinin. Loss or downregulation of SORBS2 disrupts intercellular junctions, induces aberrant cytoskeletal remodelling, and derepresses genes associated with EMT, ultimately enabling cancer cells to acquire migratory and invasive capabilities that drive metastatic progression. Thus, the loss of SORBS2 in epithelial cells represents a critical event in malignant tumour progression.

##### 3.1.1.3 Epigenetic Regulation

SORBS2 expression is finely regulated by epigenetic modifications. In ovarian cancer, the AT‐rich interactive domain‐containing protein 5B (ARID5B) and PHD finger protein 2 (PHF2) complex binds to H3K36me2‐modified chromatin regions, activating SORBS2 transcription via demethylation to inhibit metastatic colonization [41]. Conversely, in gastric cancer, HSF1 and MORC2 recruit the Polycomb repressive complex 2 (PRC2) to the promoter region of the SORBS2 homologue, silencing its expression through H3K27me3 modification and promoting gastric cancer metastasis [17]. In gliomas, the super‐enhancer RNA LINC02454 activates SORBS2 transcription by maintaining chromatin openness at the SORBS2 locus (via recruitment of the mediator complex subunit MED1). SORBS2 overexpression enhances temozolomide (TMZ)‐induced apoptosis, while its low expression correlates with chemotherapy resistance [42]. Additionally, DNA methylation analyses reveal hypermethylation of the SORBS2 promoter in cervical and colorectal cancers, leading to transcriptional silencing and driving tumour progression by derepressing the Notch pathway [43, 44].

##### 3.1.1.4 Immunological Microenvironment Regulation

SORBS2 remodels the tumour microenvironment by modulating immune cell functions and adhesion signalling. In ovarian cancer, SORBS2 stabilizes WFDC1 (WAP four‐disulfide core domain protein 1) and IL‐17D mRNAs, inhibiting metastatic niche formation and blocking monocyte polarization into pro‐tumorigenic myeloid‐derived suppressor cells (MDSCs) and M2 macrophages, thereby fostering an anti‐tumour immune milieu [45]. In osteosarcoma, low SORBS2 expression significantly correlates with increased mast cell infiltration in tumours. Its loss may promote angiogenesis and stromal remodelling via activation of mast cell‐associated pathways (e.g., histamine signalling), resulting in reduced disease‐free survival [46]. Moreover, in breast cancer models, p38α kinase transcriptionally represses SORBS2, weakening cell‐matrix adhesion and impairing spheroid formation. SORBS2 knockout partially reverses phenotypes caused by p38α deficiency, suggesting SORBS2 is a key downstream effector of p38α in adhesion regulation [47].

#### 3.1.2 Context‐Dependent Oncogenic Roles in Specific Malignancies

##### 3.1.2.1 Pro‐Metastatic Functions: TNBC and Gastric Cancer

Although SORBS2 primarily exerts tumour‐suppressive effects in malignancies, it exhibits pro‐oncogenic properties in certain contexts. In triple‐negative breast cancer (TNBC), SORBS2 may function as an RNA‐binding protein (RBP) by binding to the coding sequence (CDS) or 3′UTR of target mRNAs, regulating pro‐metastatic gene networks (e.g., Wnt pathway), thereby driving pulmonary metastasis formation [31]. In gastric cancer, muscone treatment suppresses tumour proliferation by downregulating SORBS2 expression, while SORBS2 knockout significantly reduces cell survival and migration, suggesting its potential pro‐tumorigenic role via unknown mechanisms (e.g., metabolic reprogramming) [8]. This functional dichotomy may stem from differential expression of SORBS2‐interacting proteins within tumour microenvironments.

##### 3.1.2.2 Aberrant Fusion and Signal Activation

Chromosomal translocations generating SORBS2 fusion proteins can aberrantly activate pro‐oncogenic signalling. In acute myeloid leukaemia (AML‐M5), the MLL‐SORBS2 fusion protein retains the SH3‐C domain of SORBS2, binds to CBL (E3 ubiquitin ligase) and ABL tyrosine kinase, and inhibits CBL‐mediated ubiquitination and degradation of ABL. This results in sustained ABL activation, driving leukaemic cell proliferation and survival [48]. In pancreatic acinar cell carcinoma (PACC), the BRAF::SORBS2 fusion protein may activate the Mitogen‐activated protein kinase (MAPK) pathway via retained BRAF kinase domains, though its precise regulatory mechanisms require further validation [49].

##### 3.1.2.3 Microenvironment‐Dependent Pro‐Invasive Effects

In glioblastoma, high SORBS2 expression correlates significantly with poor patient prognosis (median survival: 10.6 months). Mechanistically, phosphodiesterase 1C (PDE1C) downregulates cAMP levels to activate downstream effectors, inducing SORBS2 expression and promoting tumour cell adhesion and invasion. Clinical data analyses reveal that SORBS2 overexpression is closely associated with enrichment of cell adhesion pathways (e.g., integrin signalling) [37]. Additionally, in trophoblasts, SORBS2 enhances glycolysis by stabilizing hexokinase 2 (HK2) mRNA to promote cell migration [30], suggesting a similar metabolic reprogramming mechanism may support invasive phenotypes in tumours.

#### 3.1.3 Clinical Correlations: Prognostic Value and Therapeutic Potential

SORBS2 expression levels are closely linked to patient prognosis and therapeutic responses. In hepatocellular carcinoma, bladder cancer and osteosarcoma, low SORBS2 expression indicates advanced tumour stage, high metastatic risk and reduced survival, with its status as an independent prognostic factor incorporated into multi‐gene predictive models [50, 51, 52]. In radiation‐induced paediatric thyroid cancers, SORBS2 expression is 3.01‐fold lower than in sporadic tumours, with no DNA copy number alterations, implicating epigenetic silencing as an early event in radiation‐associated carcinogenesis [53]. Therapeutically, targeting SORBS2 regulatory networks (e.g., the LINC02454‐SORBS2 axis) enhances glioblastoma sensitivity to temozolomide (TMZ) [42], while disrupting MLL‐SORBS2 fusion protein interactions with ABL may offer novel treatment strategies for AML [48].

### 3.2 Cardiovascular Diseases

**FIGURE 4:**
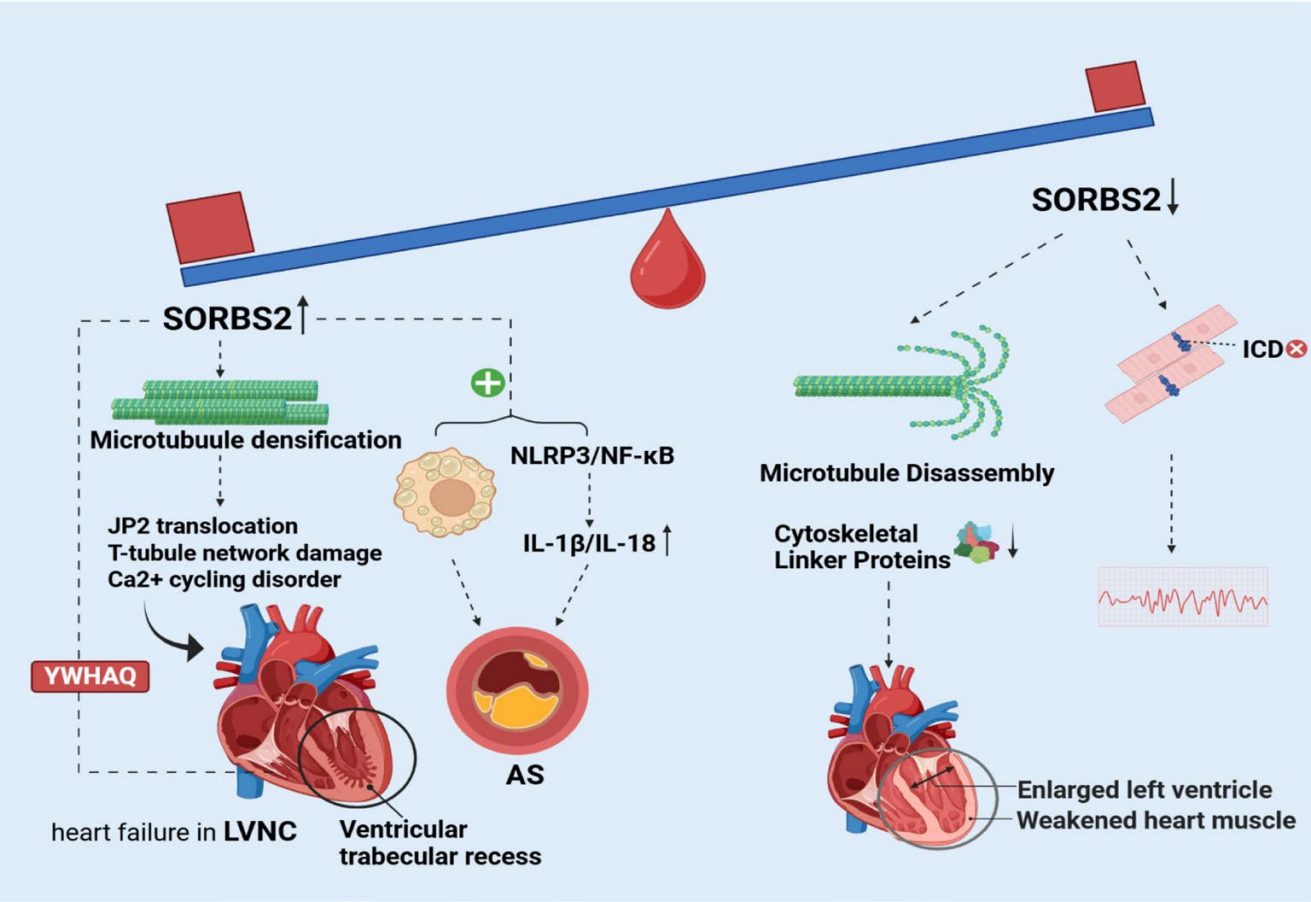
The role of SORBS2 in cardiovascular diseases. SORBS2 exhibits dose‐dependent effects in the cardiovascular system: Reduced SORBS2 expression disrupts cardiomyocyte cytoskeletal integrity, resulting in microtubule depolymerisation and aberrant intercellular junctions. It concurrently disrupts intercalated disc (ICD) structure, contributing to arrhythmogenesis. Overexpression of SORBS2 protein can cause excessive polymerization of β‐tubulin, leading to abnormal ventricular development. It also promotes the occurrence of inflammation and the formation of foam cells, thereby participating in the pathological process of AS.

SORBS2 is widely expressed in cardiomyocytes, fibroblasts, and vascular smooth muscle cells, participating in the pathogenesis of various cardiovascular diseases through regulation of cytoskeletal dynamics, signal transduction and intercellular communication. Recent studies have elucidated its critical roles in structural heart disease, myocardial infarction, inherited cardiac disorders, atherosclerosis and arrhythmias (Figure 4). The molecular mechanisms underlying these roles are detailed below from distinct disease perspectives.

#### 3.2.1 Structural Heart Disease and Heart Failure: Cytoskeletal Integrity and Compensatory Mechanisms

Structural heart disease encompasses a spectrum of conditions associated with anatomical abnormalities of the heart and adjacent great vessels, which may be congenital or acquired. Myocardial hypertrophy represents an adaptive response to pressure overload, but chronic pathological hypertrophy can progress to heart failure. Bang et al. [54] first discovered that cardiac fibroblasts secrete miR‐21‐3p (previously known as miR‐21*) through exosomes, targeting and inhibiting the expression of SORBS2 and its homologous protein PDLIM5 in cardiomyocytes, thereby inducing cardiomyocyte hypertrophy. McLendon et al. [35] generated cardiomyocyte‐specific Sorbs2 knockout mice (*Sorbs2*‐CKO), which developed left ventricular dilation, systolic dysfunction and fatal heart failure in adulthood. Mechanistically, SORBS2 deficiency causes aberrant microtubule polymerization and dysregulated expression of cytoskeletal linker proteins (e.g., α‐actinin, desmin), disrupting cardiomyocyte mechanical stability and ultimately leading to dilated cardiomyopathy (DCM). Notably, SORBS2 expression is markedly elevated in heart failure patients and murine models, correlating positively with ejection fraction (EF), myocardial hypertrophy and fibrosis severity, suggesting its upregulation may exert a compensatory role by preserving cytoskeletal integrity to delay remodelling [55].

#### 3.2.2 Myocardial Infarction: Dynamic Expression and Cardioprotective Role

Myocardial infarction (MI), characterized by abrupt reduction or complete cessation of coronary blood flow resulting in myocardial ischaemia, hypoxia and necrosis, is a severe cardiovascular event [56]. Post‐MI, SORBS2 exhibits spatiotemporal expression dynamics. Kakimoto et al. [57] identified via proteomic profiling that SORBS2 protein levels plummet in infarcted myocardial tissue of MI patients, while serum SORBS2 concentrations rise significantly, implicating it as a potential early biomarker of myocardial injury. Mechanistic studies targeting SORBS2 may offer novel cardioprotective strategies. In the MI mouse model, the expression of SORBS2 in myocardial cells of the peri‐infarct zone was significantly upregulated at 7 days post‐operation, and this process was regulated by the transcription factor GATA4 activated by mechanical stress. SORBS2 modulates integrin‐cytoskeletal linkages via WAVE complex interactions, influencing cardiomyocyte adhesion and extracellular matrix (ECM) remodelling to mediate tissue repair or fibrosis [55]. This dynamic expression pattern indicates SORBS2 may spatially coordinate the balance between myocardial repair and pathological remodelling.

#### 3.2.3 Inherited Cardiac Disorders: 4q Deletion Syndrome and Left Ventricular Non‐Compaction

SORBS2 mutations or dysregulation are closely linked to inherited cardiac diseases. Liang et al. [58] reported that SORBS2 haploinsufficiency in 4q deletion syndrome patients causes second heart field (SHF) developmental defects, manifesting as atrial septal defects or double atrial septum (DAS). This involves disrupted NOTCH and SHH signalling, leading to imbalanced proliferation and differentiation of cardiac progenitor cells. Additionally, SORBS2 is specifically upregulated in left ventricular non‐compaction cardiomyopathy (LVNC) myocardial tissue—distinct from most structural heart diseases. Further studies revealed that in LVNC, overexpressed SORBS2 binds β‐tubulin to promote excessive polymerization, resulting in hyperdense microtubule networks, aberrant junctophilin‐2 (JP2) distribution, T‐tubule disorganization, and ultimately disrupted calcium homeostasis, impaired contractility and heart failure [59]. Li et al. [36] further demonstrated that SORBS2 interaction with YWHAQ (tyrosine 3‐monooxygenase/tryptophan 5‐monooxygenase activation protein theta, a 14‐3‐3 protein family member) suppresses cardiomyocyte cell cycle progression, causing developmental arrest and exacerbating LVNC phenotypes. These findings underscore SORBS2's dual roles in cardiac development and structural maintenance.

#### 3.2.4 Atherosclerosis: Inflammasome Activation and Foam Cell Formation

Atherosclerosis (AS), a primary contributor to cardiovascular events, involves SORBS2 in its pathogenesis through regulation of inflammatory responses and foam cell formation. Liu et al. [60] demonstrated that SORBS2 silencing attenuates OxLDL‐induced inflammation by inhibiting the NLRP3 inflammasome and NF‐κB signalling pathways. Specifically, SORBS2 knockdown reduced NLRP3 expression, cleaved caspase‐1 (p20) levels, and the secretion of IL‐1β and IL‐18. Furthermore, SORBS2 silencing decreased reactive oxygen species (ROS) production—a well‐established trigger for NLRP3 activation—and suppressed the assembly of ASC specks, a hallmark of inflammasome activation. Concurrently, SORBS2 silencing promoted cholesterol efflux via the PPARγ/ABCG1 pathway, thereby reducing foam cell formation [61, 62, 63]. These findings collectively indicate that SORBS2 acts as an upstream driver of NLRP3 inflammasome activation by promoting ROS production and NF‐κB signalling, thereby exacerbating inflammatory responses and foam cell formation in atherosclerosis. In summary, SORBS2 plays a dual role in macrophages by promoting both inflammation and foam cell formation, positioning it as a central node linking metabolic dysregulation to vascular inflammation. Additionally, in rat models, extracellular vesicles (EVs) derived from vascular fibroblasts were found to carry miR‐21‐3p, which suppresses SORBS2 expression in vascular smooth muscle cells (VSMCs), promoting VSMC proliferation and migration and exacerbating vascular remodelling [64]. This finding suggests that SORBS2 may have broader functions within vascular wall cells, Future studies employing single‐cell spatial omics and cell‐specific genetic manipulation models are warranted to systematically dissect the role of SORBS2 in endothelial cells, particularly its potential involvement in early atherosclerotic events such as vascular permeability and leukocyte adhesion.

#### 3.2.5 Arrhythmias: Ion Channel Dysregulation and Intercalated Disc Disruption

SORBS2 deficiency can induce arrhythmias by disrupting ion homeostasis and intercellular connections. Qian et al. [65] observed that Sorbs2 knockout murine ventricular cardiomyocytes exhibit prolonged action potential duration, depolarised resting membrane potential, and spontaneous ventricular arrhythmias, attributed to dysregulated expression and function of sodium channels (Nav1.5), L‐type calcium channels (Cav1.2), and inward rectifier potassium channels (Kir2.1). Further studies revealed that SORBS2 maintains intercalated disc (ICD) structural integrity and regulates connexin 43 (Cx43) expression, preventing right ventricular dilation and arrhythmogenic cardiomyopathy (ACM). Rare SORBS2 splice variants have been identified in ACM cases, highlighting its potential as a novel susceptibility gene [66]. Moreover, SORBS2 interacts with microtubules to influence ion channel membrane localisation; its loss disrupts channel protein distribution, exacerbating arrhythmia risk [6].

### 3.3 Metabolic Diseases

**FIGURE 5:**
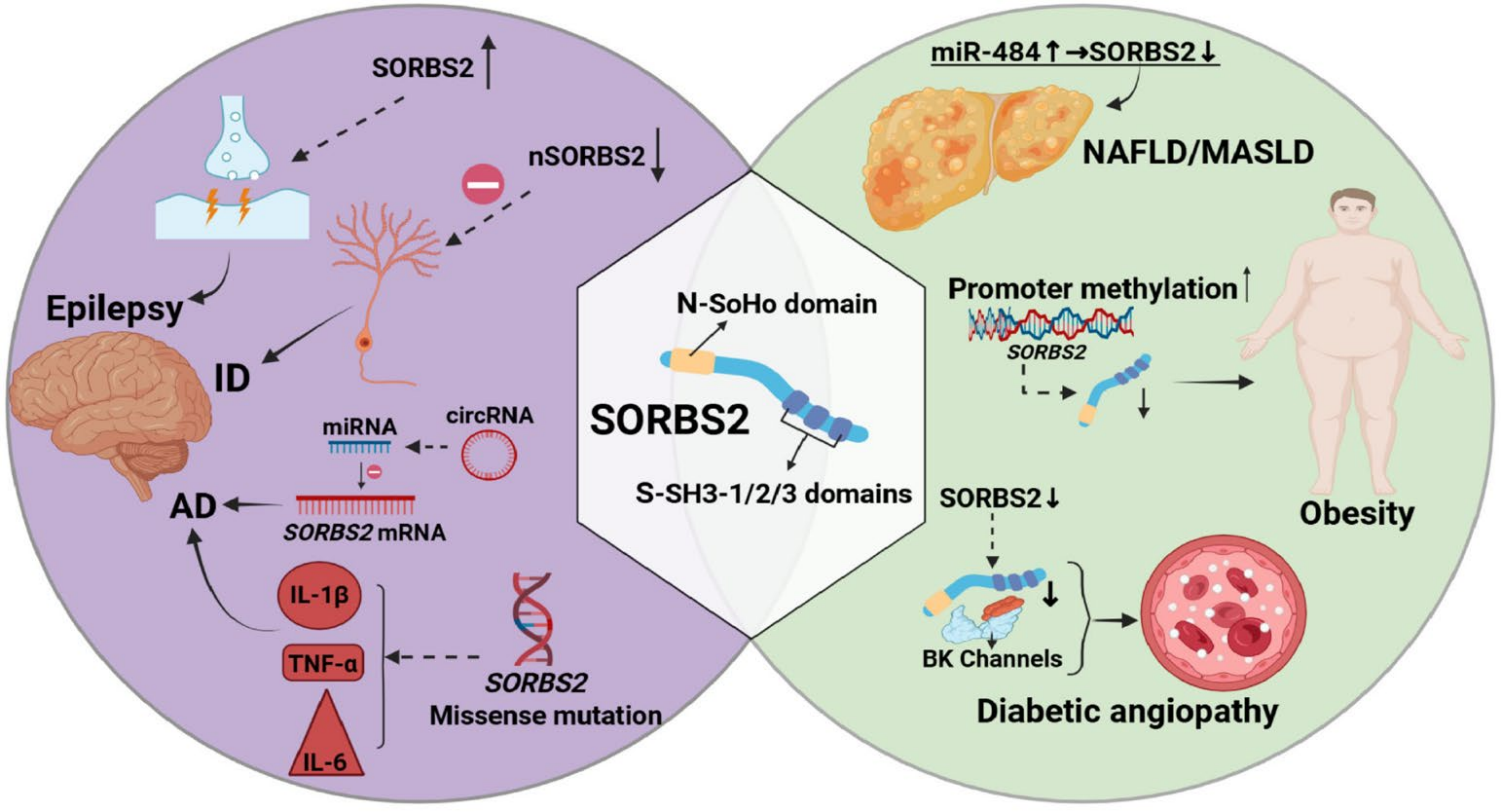
The role of SORBS2 in metabolic and neurological disorders. The left panel illustrates SORBS2 contributing to the pathogenesis of temporal lobe epilepsy, intellectual disability and Alzheimer's disease via regulation of excitatory synaptic transmission, dendritic development and inflammatory responses. The right panel depicts the regulation of SORBS2 expression by miR‐484 and promoter methylation, while also illustrating its association with non‐alcoholic fatty liver disease (NAFLD), obesity and diabetic vasculopathy. MASLD: Metabolic Dysfunction‐Associated Steatotic Liver Disease. (MASLD constitutes the updated and replacement terminology for NAFLD).

Previous research on SORBS2 primarily focused on its roles in cancer and cardiovascular diseases. In recent years, SORBS2 has been demonstrated to play critical roles in the pathophysiological processes of various metabolic diseases. Through its regulation of signalling pathways, epigenetic modifications, and RNA‐binding activity, SORBS2 participates in the progression of diabetes mellitus, obesity, NAFLD, and other metabolic disorders, positioning it as a focal point for cross‐disease research (Figure 5).

#### 3.3.1 Diabetic Vascular Complications: BK Channel Dysfunction and Nrf2 Signalling

Diabetic vascular complications, a common and severe health issue in diabetic patients, are categorized into microvascular and macrovascular complications. These complications primarily arise from vascular damage induced by chronic hyperglycaemia [67]. However, recent studies reveal that SORBS2 deficiency can independently cause vascular dysfunction irrespective of metabolic abnormalities (e.g., hyperglycaemia, obesity). Sun et al. [23] discovered that in diabetic mouse models, Nrf2 signalling failure leads to a significant downregulation of SORBS2 expression, resulting in impaired BK channel function and coronary vasculopathy. Further investigations demonstrated that SORBS2 binds to the α and β1 subunits of BK channels via its SH3 and SoHo domains, maintaining channel Ca^2+^ sensitivity and vasodilatory capacity. This confirms SORBS2's direct involvement in the molecular mechanisms underlying diabetic vascular complications. This study is the first to elucidate SORBS2's role in diabetic vasculopathy through the Nrf2‐BK pathway, bridging a gap in the molecular understanding of BK channelopathies in diabetes and providing a theoretical foundation for targeted therapies.

#### 3.3.2 Obesity: Promoter Methylation and Adipocyte Dysfunction

In obesity, epigenetic modifications of SORBS2 are closely linked to adipose tissue metabolic dysregulation. Keller et al. [11] identified through genome‐wide DNA methylation and transcriptomic analyses that promoter methylation of SORBS2 is significantly elevated in visceral adipose tissue of obese individuals. In vitro experiments confirmed that this methylation directly suppresses gene expression, potentially contributing to lipid metabolism disorders and obesity progression. The methylation level of this gene exhibits a significant correlation with body mass index (BMI), suggesting its role as a key epigenetic regulatory target in obesity. A systematic review by Gharipour et al. [68] further revealed that promoter methylation of SORBS2 may participate in obesity development by regulating insulin‐mediated glucose transporter type4 (GLUT4) translocation and adipocyte differentiation abnormalities. Concurrently, its downregulated expression associates with neuropathological mechanisms of mood disorders (e.g., depression) via Notch signalling‐mediated synaptic plasticity impairment and inflammatory responses.

#### 3.3.3 NAFLD: miR‐484‐Mediated Lipid Metabolism

NAFLD is a clinical‐pathological syndrome characterized by excessive fat deposition in hepatocytes, excluding alcohol consumption and other well‐defined liver‐damaging factors. SORBS2 exerts regulatory effects on NAFLD progression through miRNA‐mediated lipid metabolism modulation. Jia et al. [10] demonstrated that miR‐484 directly targets the 3′‐UTR specific region of SORBS2 mRNA, downregulating its expression, suppressing mitochondrial β‐oxidation, and promoting hepatic lipid accumulation. Knockout of miR‐484 restores SORBS2 activity, ameliorating hepatic steatosis and inflammation in high‐fat diet‐fed mice. These findings highlight SORBS2 as a potential therapeutic target for NAFLD.

### 3.4 Neurological and Psychiatric Disorders

Recent studies have revealed that abnormal expression or genetic variations of SORBS2 are closely associated with multiple neurological and psychiatric disorders, including temporal lobe epilepsy (TLE), Alzheimer's disease (AD), intellectual disability (ID), and bipolar disorder (Figure 5). The following sections detail its specific roles and mechanisms in these diseases.

#### 3.4.1 Temporal Lobe Epilepsy: AMPAR‐Mediated Excitatory Transmission

Temporal lobe epilepsy (TLE) is a common drug‐refractory epilepsy type, with pathogenesis linked to abnormal excitatory synaptic transmission [69]. Ban et al. [70] investigated kainic acid (KA)‐induced TLE mouse models and brain tissues from TLE patients, revealing significant upregulation of SORBS2 in the hippocampus and cortex. Further experiments demonstrated that downregulating hippocampal SORBS2 prolongs the latency of spontaneous recurrent seizures (SRSs) and reduces seizure frequency. Mechanistically, SORBS2 enhances excitatory synaptic transmission in CA1 pyramidal neurons by regulating the expression of AMPAR (α‐amino‐3‐hydroxy‐5‐methyl‐4‐isoxazolepropionic acid receptor) subunits GluA1 and GluA2, thereby promoting the occurrence of epilepsy. This study is the first to elucidate SORBS2's role in driving TLE progression via the AMPAR pathway, offering novel insights into anti‐epileptic therapies targeting SORBS2.

#### 3.4.2 Alzheimer's Disease: PSEN1 Mutation and ceRNA Networks

Alzheimer's disease (AD) is a neurodegenerative disorder characterized by progressive cognitive decline. In AD, SORBS2 has been identified as a critical gene influencing disease onset and pathological progression. Lee et al. [71] conducted a genome‐wide association analysis on a Caribbean AD pedigree carrying the presenilin 1 (*PSEN1*) p.G206A mutation, identifying SORBS2 as a genetic modifier of age at onset that significantly affects the timing of early‐ and late‐onset AD. Subsequent research further revealed that circular RNA (circRNA)‐associated competing endogenous RNA (ceRNA) networks regulate SORBS2 expression levels in AD pathology. Ma et al. [72] found through ceRNA network analysis that SORBS2 expression is suppressed by miRNAs such as miR‐219b‐5p, and its dysregulation may directly contribute to AD‐related cognitive decline and pathological progression by impairing synaptic plasticity and neuronal connectivity [72]. Additionally, Wang et al. [73] identified a SORBS2 missense mutation (T189M) in a Han Chinese early‐onset AD pedigree. This mutation induced cognitive deficits in transgenic mice and exacerbated neuroinflammation and neuronal damage by activating microglia, upregulating pro‐inflammatory cytokines (IL‐1β, IL‐6 and TNF‐α), and increasing the Aβ42/Aβ40 ratio.

#### 3.4.3 Intellectual Disability: nSORBS2 and Dendritic Development

Intellectual disability (ID) is a common neurodevelopmental disorder strongly associated with genetic defects and environmental factors [74, 75]. Microdeletions/microduplications in the chromosomal 4q35.1 region are linked to ID, and SORBS2, located in this region, is hypothesised to be a potential pathogenic gene [32]. Copy number variations of SORBS2 are closely associated with ID. Zhang et al. [33] constructed a SORBS2 knockout mouse model and observed reduced dendritic complexity in hippocampal granule cells and decreased frequency of postsynaptic excitatory currents. Behavioural experiments revealed long‐term object recognition memory deficits, contextual fear memory impairment, and attenuated acoustic startle responses in knockout mice. Mechanistically, the neuron‐specific isoform nSORBS2, encoded by SORBS2, co‐localizes with F‐actin to regulate dendritic development and synaptic function. This study is the first to elucidate the neurobiological basis of SORBS2 deficiency in ID.

#### 3.4.4 Psychiatric Disorders: Synaptic Actin Dynamics and Bipolar Disorder‐Like Phenotypes

Research on SORBS2 in psychiatric disorders highlights its central role as a synaptic scaffolding protein. Studies indicate that SORBS2‐deficient mice exhibit manic/bipolar disorder‐like phenotypes, with synaptic actin homeostasis imbalance proposed as a key pathological mechanism [33, 34]. Gene knockdown experiments showed that SORBS2 depletion significantly reduces mushroom spine density, induces abnormal filopodia proliferation, and disrupts excitatory synapse localisation to dendritic spines, forcing their relocation to dendritic shafts. Mechanistically, SORBS2 negatively regulates Rac1/PAK/WAVE1 signalling pathway activity by binding actin regulators such as WAVE1 via its SH3 domain, thereby maintaining dynamic equilibrium of the synaptic actin network [34]. Furthermore, SORBS2 interacts with autism‐associated protein Shank3 and fragile X syndrome‐related protein CYFIP2, suggesting its role as a molecular hub in multiple neurodevelopmental disorders [76, 77, 78]. Epigenetic studies further identified differential methylation in SORBS2 enhancer regions as correlating with antidepressant treatment response, implicating its role in modulating emotion‐related neural plasticity and disease outcomes [79].

## 4 Cross‐Disease Potential Association of SORBS2

Although the roles of SORBS2 in cancer, cardiovascular diseases, metabolic disorders and neurological diseases have been partially elucidated, its cross‐disease interactions and shared regulatory mechanisms remain underexplored. This section focuses on SORBS2 as a ‘molecular hub’ with cross‐disease properties, dissecting its potential framework for linking multisystem pathological processes through inflammation, epigenetic modifications and metabolic reprogramming.

### 4.1 Molecular Link Between NAFLD and Hepatocellular Carcinoma

Based on previous studies and analyses of GEO databases (GSE162694, GSE135251), SORBS2 expression is downregulated in both NAFLD‐affected liver tissues and HCC. In NAFLD, SORBS2 suppresses disease progression by regulating lipid metabolism and inflammation, while in HCC, it exerts tumour‐suppressive effects via the RORA and c‐Abl/ERK pathways [80, 81]. Notably, NAFLD—particularly non‐alcoholic steatohepatitis (NASH)—is a major risk factor for HCC [82]. We hypothesise that SORBS2 may serve as a key molecular nexus connecting NAFLD and HCC, where its sustained downregulation in NAFLD could drive chronic inflammation, fibrosis and genomic instability, significantly elevating HCC risk. Regulatory mechanisms of SORBS2 expression differ between these conditions: in NAFLD, miR‐484 upregulation inhibits SORBS2, promoting steatosis [10], whereas in HCC, miR‐484 may similarly exacerbate tumour progression (though direct evidence is lacking, miRNA‐mediated cross‐disease regulation is well‐documented). Similarly, Myocyte enhancer factor 2D (MEF2D), which is overexpressed in HCC, suppresses SORBS2 transcription by binding to its promoter. This mechanism may already reduce SORBS2 during the NAFLD stage, forming a continuous regulatory axis from metabolic dysregulation to tumorigenesis [50]. Future studies should validate the dynamic regulation of SORBS2 in NAFLD‐HCC transition and its potential as a cross‐disease therapeutic target.

### 4.2 Shared Regulatory Networks in Metabolic Diseases

Diabetes, NAFLD and obesity frequently co‐occur clinically [83, 84]. Notably, a 5%–10% weight loss significantly improves NAFLD, insulin sensitivity, and glycaemic control, while Glucagon‐like peptide‐1 (GLP‐1) receptor agonists (e.g., liraglutide) concurrently lower blood glucose, reduce weight, and ameliorate NASH, underscoring metabolic interplay among these disorders [85, 86, 87]. In all three diseases, SORBS2 acts as a shared regulatory factor, with its expression modulated by epigenetic (methylation, miRNA) and transcriptional (Nrf2) mechanisms. Whether these regulatory mechanisms are organ‐specific requires further investigation. Mitochondrial dysfunction and oxidative stress are implicated in diabetic vascular injury, adipose tissue inflammation in obesity, and hepatocyte lipotoxicity in NAFLD. We speculate that SORBS2 may influence metabolic diseases by modulating mitochondrial function or antioxidant responses [88, 89, 90]. Additionally, chronic low‐grade inflammation common to these diseases suggests SORBS2 could affect progression via inflammatory signalling pathways (e.g., NF‐κB or cytokine expression) [91, 92]. Future research should focus on SORBS2's cross‐organ regulatory networks and its links to mitochondrial function and inflammation, thereby unravelling shared pathological mechanisms in diabetes, obesity and NAFLD to transcend current single‐disease treatment limitations.

### 4.3 Cross‐Disease Roles of Inflammation and Epigenetic Regulation

SORBS2 exerts its hub function in multisystem diseases via dual mechanisms of inflammation and epigenetic regulation. In atherosclerosis, SORBS2 activates the NLRP3 inflammasome to promote foam cell formation, while in AD, its mutant variant exacerbates neuroinflammation by upregulating IL‐1β and TNF‐α, suggesting therapeutic potential in targeting the SORBS2‐NLRP3 axis across diseases [60, 73]. Furthermore, SORBS2 is not only regulated by DNA methylation and histone modifications but also acts as an RNA‐binding protein (RBP) to stabilize target mRNAs (e.g., RORA, MTUS1), thereby influencing epigenetically associated gene expression. This unique ‘epigenetic‐RNA crosstalk’ mechanism amplifies its cross‐pathological regulatory capacity in metabolic disorders, cancer and neurodegenerative diseases.

## 5 Conclusion

SORBS2, a multifunctional scaffold protein, occupies a central position in the pathogenesis of diverse human diseases. Its structural diversity, including SoHo and SH3 domains, enables dynamic interactions with signalling molecules, cytoskeletal regulators, and target mRNAs, thereby coordinating processes such as cellular signal transduction, mechanical stability and transcriptional regulation. In malignancies, SORBS2 primarily functions as a tumour suppressor by stabilizing tumour‐suppressive mRNAs (e.g., RORA, MTUS1) and remodelling the immune microenvironment to inhibit tumour progression. However, in specific cancers (e.g., triple‐negative breast cancer, gastric cancer), its role is context‐dependent, exhibiting pro‐metastatic effects. In cardiovascular diseases, the dysfunction of SORBS2 can disrupt the integrity of the cytoskeleton and the localization of ion channels, thereby leading to cardiomyopathy and arrhythmia. However, in left ventricular myocardial densification, its overexpression abnormally aggravates the excessive aggregation of microtubules. Metabolic disorders such as diabetes and NAFLD are closely linked to SORBS2's roles in mitochondrial function and lipid metabolism, which are influenced by epigenetic silencing or miRNA targeting. Crucially, SORBS2 orchestrates multisystem pathological processes through common mechanisms such as its distinct structural domains mediating protein–protein interactions and epigenetic‐RNA cross‐regulatory networks. Its cross‐disease properties as a molecular hub may provide critical entry points for novel therapeutic strategies.

## 6 Outlook

Future advancements require breakthroughs in three domains—mechanistic elucidation, technological innovation and clinical translation—to transform SORBS2 from a ‘disease nexus’ into a therapeutic target. Mechanistic depth should focus on tissue specificity and dynamic post‐translational modifications. Single‐cell spatial omics could delineate the distribution of SORBS2 splice isoforms (e.g., nSORBS2, SORBS2β) in tissues such as the heart and brain, while real‐time fluorescence tracking technologies could reveal how phosphorylation (e.g., Y573) dynamically regulates its function in myocardial ischaemia or tumour metastasis [93]. Additionally, exploring whether SORBS2 forms RNA‐protein condensates via phase separation to stabilize target mRNAs (e.g., RORA) may unveil novel RNA‐targeted therapies [94]. Clinical translation demands multi‐modal intervention strategies. Designing antisense oligonucleotides (ASOs) or miRNA antagonists (e.g., anti‐miR‐484) to restore SORBS2 expression in metabolic diseases and cancers [95], or combining SORBS2 activators with Programmed cell death protein 1 (PD‐1) inhibitors in HCC to synergistically remodel the immune microenvironment, holds promise. Concurrently, developing dynamic monitoring technologies for exosome SORBS2 or circulating miR‐484 could enable cross‐disease prognostic models to guide precision therapy. Technological innovation may leverage AI to optimize small‐molecule targeting specificity and Clustered regularly interspaced short palindromic repeats (CRISPR)‐based epigenetic editing tools to reverse SORBS2 methylation silencing [96]. Establishing a ‘SORBS2‐Disease Interaction Database’ to integrate multi‐omics data and identify shared pathways (e.g., NLRP3 inflammasome) could facilitate ‘single‐target‐multidisease’ strategies. In summary, SORBS2 research must transcend traditional paradigms, fostering interdisciplinary collaboration to achieve a ‘mechanism‐technology‐clinic’ closed‐loop. Its ‘crossroads’ nature presents both challenges and unparalleled opportunities to unlock cross‐disease therapeutic breakthroughs.

## Author Contributions

Qiwei Jia wrote the manuscript and prepared the figures. Yong Zhang revised the manuscript. Both authors read and approved the final version of the manuscript.

## Funding

The authors have nothing to report.

## Ethics Statement

The authors have nothing to report.

## Consent

The authors have nothing to report.

## Conflicts of Interest

The authors declare no conflicts of interest.

## Data Availability

The data supporting the findings of this study are available within the article. The data presented in Figure 1 were derived from The Human Protein Atlas (available online at proteinatlas.org). All references cited in this study are sourced from the PubMed public database, which provides authentic and publicly accessible data.
